# A comprehensive review on microbial hyaluronan-degrading enzymes: from virulence factors to biotechnological tools

**DOI:** 10.1186/s40643-024-00832-x

**Published:** 2024-12-25

**Authors:** Jia-Yu Jiang, Dai Xue, Jin-Song Gong, Qin-Xin Zheng, Yue-Sheng Zhang, Chang Su, Zheng-Hong Xu, Jin-Song Shi

**Affiliations:** 1 Key Laboratory of Carbohydrate Chemistry and Biotechnology of Ministry of Education, School of Life Sciences and Health Engineering, Jiangnan University, Lihu Avenue No. 1800, Wuxi, 214122 People’s Republic of China; 2 The Novo Nordisk Foundation Center for Biosustainability, Technical University of Denmark, Kemitorvet 220, 2800 Kgs, Lyngby, Denmark; 3 School of Biotechnology, Jiangnan University, Wuxi, 214122 People’s Republic of China; 4 College of Biomass Science and Engineering, Sichuan University, Chengdu, 610065 People’s Republic of China; 5 Affiliated Children’s Hospital of Jiangnan University, Wuxi, 214023 People’s Republic of China

**Keywords:** Hyaluronan-degrading enzymes, Hyaluronan lyase, Hyaluronidase, Host invasion, Disease development, Anti-bacteria, Cancer therapy, Hyaluronan degradation, Hyaluronan detection

## Abstract

Hyaluronan (HA), a natural high molecular weight polysaccharide, has extensive applications in cosmetology and medical treatment. Hyaluronan-degrading enzymes (Hyals) act as molecular scissors that cleave HA by breaking the glucosidic linkage. Hyals are present in diverse organisms, including vertebrates, invertebrates and microorganisms, and play momentous roles in biological processes. In recent years, microbial Hyals (mHyals) have gained considerable attention for their exceptional performance in the production and processing of HA. Moreover, the applications of mHyals have been greatly extended to various biomedical fields. To explore the potential applications of mHyals, a thorough comprehension is imperative. In this context, this review systematically summarizes the sources, structures, mechanisms and enzymatic properties of mHyals and discusses their biological functions in host invasion, disease development, and regulation of intestinal flora. Furthermore, versatile applications inspired by their biological functions in medicine development, molecular biology, and industrial biotechnology are comprehensively reviewed. Finally, prospects are presented to emphasize the importance of exploration, expression and characterization of mHyals and the necessity of excavating their potential in biotechnological fields.

**Graphical abstract:**

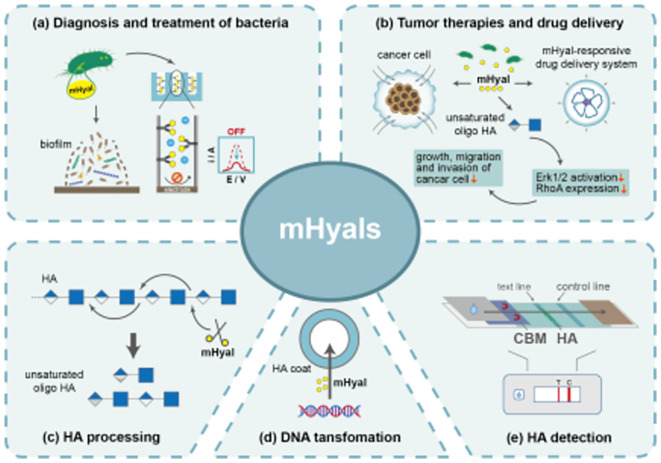

## Introduction

Hyaluronic acid (HA), also known as hyaluronan, is a linear and highly charged glycosaminoglycan (GAG) comprised of repetitive disaccharide units consisting of D-gluconic acid (GlcA) and N-acetylglucosamine (GlcNAc) (Hargittai et al. 2008). HA can be observed in the extracellular matrix (ECM) of all vertebrates as well as in the bacterial capsule of certain strains, exerting indispensable roles in biological processes within organisms owing to its exceptional structural characteristics (Noble 2002). The functions of HA fragments are highly reliant on the molecular mass of polymers (Stern et al. 2006). In recent years, there has been increasing attention towards low-molecular-weight HAs (LMW-HAs) and HA oligosaccharides (O-HAs) for their involvement in anti-inflammatory response (Olsson et al. 2018), chronic wound healing (King et al. 2020), and facilitation of angiogenesis/osteoanagenesis (Vinukonda et al. 2016) For this reason, hyaluronan-degrading enzymes (Hyals) have attracted attention and significance as indispensable and specific instruments for HA depolymerization (Buhren et al. 2016; Pavan et al. 2016).

Hyal is an enzyme that predominately degrades HA, which was initially discovered in extracts of mammalian testis and certain bacterial filtrates (Chaik et al. 1939). To date, Hyals have been mined and identified in various organisms, including vertebrates, invertebrates and microorganisms. Based on sequence similarity, Hyals are classified into the polysaccharide lyase (PL) families 5, 8, 16, 30, 33 and glycosyl hydrolase (GH) families 16, 56, 79 and 84 in the CAZy database (Sindelar et al. 2021). Mammalian and venom Hyals are members of GH56 family; they break down β-1,4-glycosidic bonds of HA to yield tetrasaccharides as the main product with N-GlcNAc at the reducing terminal and show transglycosylation properties under specific conditions (El-Safory et al. 2010). Leech Hyals belong to GH79 family and degrade HA by cleaving β-1,3-glycosidic bonds, generating tetra- and hexasaccharides as final products, with GlcA located at the reducing terminal (Lv et al. 2016). Differently, microbial Hyals (mHyals) belong to multiple families. Bacterial PL8 Hyals and PL16 Hyals mainly from bacteriophages split β-1,4-glycosidic bonds of HA by a β-elimination mechanism, furnishing predominantly unsaturated disaccharides (∆HA2) with double bonds formed between C4 and C5 on the GlcA residue (Stern et al. 2006; Stern and Jedrzejas 2006). A few Hyals from fungi have been discovered and identified as members of the GH16 family (Bakke et al. 2011). With the continuous advancement of research, novel Hyals have been discovered and ceaselessly enrich the repertoire of enzymes. Pang et al. described the enzymatic preparation methods for LMW-HAs and O-HAs, highlighting the significant potential of Hyals in eco-friendly and large-scale production of functional HA products (Pang et al. 2022).

The applications of Hyals in medical and cosmetic fields have been reviewed in recent years. Among these, mammalian Hyals, which are the most widely applied, play crucial roles in promoting uniform diffusion of the subcutaneous drugs (Khan et al. 2018; Mohankumar et al. 2023) as well as reversing complications caused by aesthetic HA injection (Borzabadi-Farahani et al. 2023; Xiao et al. 2024). Additionally, biochemical properties and potential applications of venom Hyals also have been comprehensively provided (Bordon et al. 2015; de França et al. 2023). However, the functions and applications of mHyals are often overlooked. Therefore, in this review, a phylogenetical analysis was conducted on the sequences of Hyals from microorganisms, with particular emphasis on those newly discovered. Furthermore, catalytic mechanism and enzymatic properties of mHyals were also summarized in details so as to increase the understanding of mHyals. Additionally, biological functions and applications of mHyals were specially discussed for the first time, elucidating their roles as “virulence factors” in interactions with other organisms and highlighting their versatile applications in biotechnological fields. In the end, future prospects were proposed based on the existing research. This review provides a comprehensive depiction of mHyals, which is significant for their exploration and development.

## Classification and mechanism of mHyals

**Fig. 1:**
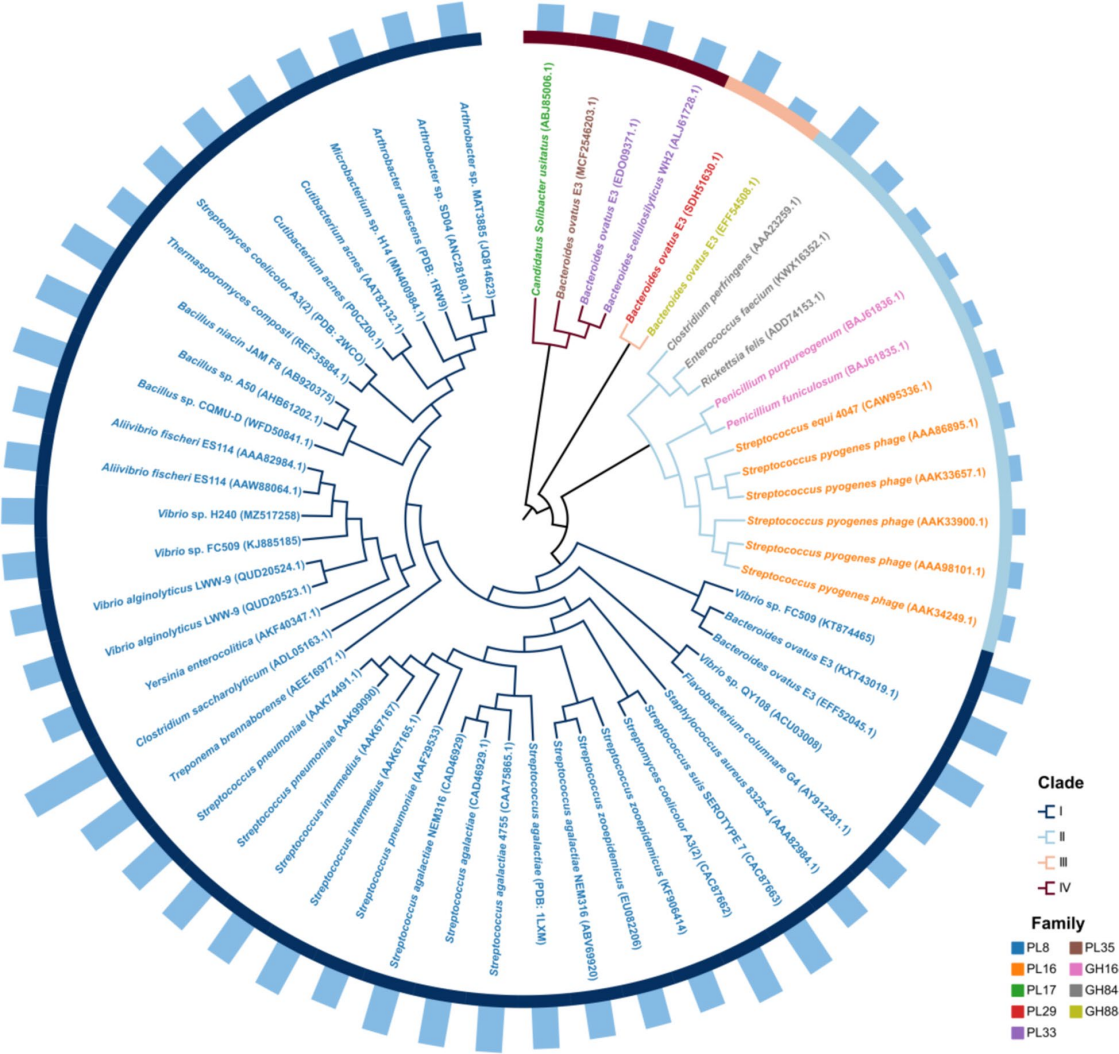
Phylogenetic analysis of mHyals. The phylogram was generated using the neighbor-joining algorithm implemented in MEG6 software and visualized by the tvBOT tool (Xie et al. 2023). Different clades and families are distinguished by distinct colors, and column heights represent sequence lengths

mHyals are widely distributed in nature, including bacteria, fungi, and bacteriophages. The evolutionary relationship of mHyals from different species is illustrated in Fig. 1.

The phylogenetic tree is primarily classified into four major evolutionary branches: PL8 in clade I; PL16, GH16, GH84 in clade II; PL29, GH88 in clade III; and PL17, PL33, PL35 in clade IV. GH16 mHyals from fungi and PL16 mHyals from bacteriophages are exclusively found within clade II, while the rest are all identified in bacteria. In comparison to mHyals located in clade I, those belonging to clade II, III and IV exhibit shorter lengths ranging from 315 to 1042 amino acids. Notably, the length of PL8 mHyals varies significantly: the mHyal from *Vibrio* sp. QY108 possesses 700 amino acids (Zhang et al. 2020), whereas the mHyal from *Clostridium saccharolyticum* WM1 consists of 1840 amino acids.

**Fig. 2:**
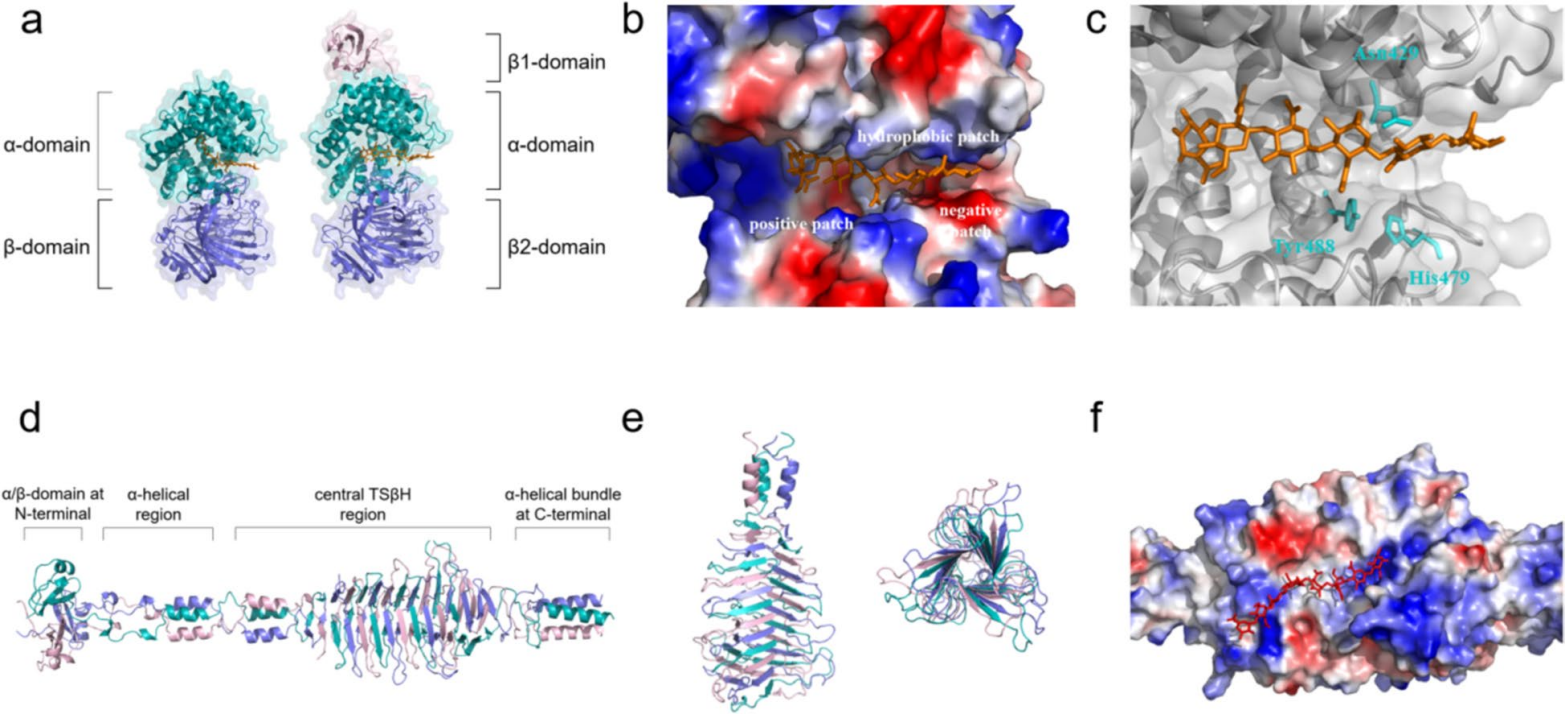
Three-dimensional structures of mHyals. **a** Structures of HA lyases from *S. pneumoniae* (left, PDB: 1LOH) and *S. agalactiae* (right, PDB: 1LXM) in complex with HA6, respectively. **b** Electrostatic potential distribution in the catalytic cleft of 1LXM. **c** Structure arrangement of the catalytic triad and HA6 substrate in the cleft of 1LXM. **d** Structures of HylP1 from *S. pyogenes* SF370 phage (PDB: 2C3F). **e** Structure of TSβH region of 2C3F. **f** Electrostatic potential distribution at the concave groove of 6X3M in complex with HA8

The majority of well-characterized mHyals belong to PL8 and PL16 families. In early investigations, these enzymes were initially treated as “virulence factors” due to their involvement in the invasion of pathogens/viruses within hosts (Li et al. 2000; Smith et al. 2005). The exploration of their structures and catalytic mechanisms has significantly contributed to the development of inhibitors targeting pathogenic bacteria and viruses. Afterwards, applications based on their biological functions have progressively emerged, elevating mHyals as a pivotal tool in the realm of biology. Interestingly, although mHyals of the PL8 and PL16 families have significant differences in structural characterization (Fig. 2), they share a common mechanism described as proton acceptance and donation. In this process, enzymes accept a proton from the substrate HA while simultaneously donating one back to complete degradation (Li et al. 2001). Specifically, C5 of HA transfers one proton to the enzyme while at the same time, the enzyme donates one proton to O4 of HA. This results in rehybridization of carbon atoms C4 and C5 from sp^3^ to sp^2^. Consequently, unsaturation occurs in the product through cleavage of β-1,4 glycosidic linkage between GlcNAc and GlcA with formation of double bonds between these two sp^2^ hybridized carbon atoms (C4 and C5) (Mello et al. 2002). In the final step, the enzyme releases its captured proton and attains a proton from the surrounding water microenvironments, thereby reverting to its initial state and becoming primed for subsequent catalysis (Li et al. 2001). The protein functions as a proton exchanger, fostering the transfer of a pair of protons between water molecules and the HA substrate, thereby inducing substrate degradation and product unsaturation.

### PL8 mHyals

PL8 family harbors the largest number of mHyals, which are exclusively found in bacteria, encompassing both gram-positive and gram-negative bacteria. It has been reported that mHyals are implicated in the infection process of pathogenic bacteria. Some gram-positive bacteria, such as *Streptococcus agalactia*e (Coleman et al. 2023), *Staphylococcus aureus* (Hart et al. 2013) and *Clostridium perfrengens* (Osman et al. 2023) secret mHyals as virulence factors to invade and penetrate tissues, facilitating bacteria dissemination. Moreover, these intruders can harness HA as a valuable carbon source for their metabolism and proliferation (Hirayama et al. 2009).

The mHyals from *Streptococcus* sp. have been extensively investigated and several crystal structures are available in the PDB database (Table 1). The mHyals of *Streptococcus pneumoniae* (spnHL) consist of two distinct structural domains: an α-helix domain (α-domain) at the N-terminal and a β-sheet domain (β-domain) at the C-terminal, connected by a short linker polypeptide (Li et al. 2000) (Fig. 2a). Both domains exhibit spherical shapes and comparable sizes. The α-domain is predominantly composed of thirteen α-helices, with ten of them organized into a twisted (α5/α5)-barrel architecture, while the β-domain, consists of five anti-parallel β-sheets formed by twenty-four β-strands (Ponnuraj et al. 2000). The larger end of this twisted barrel faces towards the β-domain, forming a profound and elongated cleft that harbors the substrate-binding site and catalytic residues crucial for HA degradation process (Jedrzejas et al. 2002).

**Table 1:** Overview of the determined crystal structures of mHyals in the PDB database

Entry code	Source	Name	Structure	Family	Refs.
1EGU	Streptococcus pneumoniae	SpnHL	SpnHL native	PL8	Li et al. (2000)
1C82	Streptococcus pneumoniae	SpnHL	SpnHL-HA2		Ponnuraj et al. (2000)
1F9G	Streptococcus pneumoniae	SpnHL	SpnHL-Vc		Li et al. (2001)
1LXK 1LOH	Streptococcus pneumoniae	SpnHL	SpnHL-HA4 SpnHL-HA6		Joshi et al. (2009)
1N7O 1N7N 1N7P 1N7Q 1N7R	Streptococcus pneumoniae	SpHyal	SpHyal-F343V SpHyal-W292A SpHyal-W292A/F343V SpHyal-W291A/W292A-HA6 SpHyal-W291A/W292A/F343V-HA6		Nukui et al. (2003)
1OJM 1OJN 1OJO 1OJP	Streptococcus pneumoniae	–	WT-∆Di0S Y408F-∆Di4S Y408F-∆Di6S WT-∆Di6S		Rigden et al. (2003)
1W3Y	Streptococcus pneumoniae	SpnHL	SpnHL-Vcpal		Botzki et al. (2004)
2BRV 2BRW	Streptococcus pneumoniae	SpnHyal	SpnHyal-PEGMME SpnHyal-MALONATE		Rigden et al. (2006a, b)
2BRP	Streptococcus pneumoniae	SpnHL	SpnHyal-phenylindole		Rigden et al. (2006a, b)
1F1S 1I8Q	Streptococcus agalactiae	SagHL	SagHL native SagHL-∆HA2		Li et al. (2001); Li and Jedrzejas (2001)
1LXM	Streptococcus agalactiae	SagHL	SagHL-HA6		Mello et al. (2002)
6WV2 6X3M	Streptococcus pyogenes phage H4489A	HylP	HylP-truncated HylP-∆HA8	PL16	–
2C3F	Streptococcus pyogenes SF370 phage	HylP1	HylP1-native		Smith et al. (2005)
2YW0 3EKA 2YVV	Streptococcus pyogenes phage 10,403	HylP2	HylP2-native HylP2-ascorbic acid HylP2-lactose		Mishra et al. (2009)
2WH7 2WB3	Streptococcus pyogenes SF370 phage	HylP2 HylP3	HylP2-truncated HylP3-truncated		Martinez-Fleites et al. (2009)
4UFQ	Streptomyces koganeiensis ATCC31394	Hyal_Sk	Hyal_Sk-native		Messina et al. (2016)
4D0Q	Streptococcus pneumoniae	SpnHL	–	CBM70	Suits et al. (2014)

The surface of the cleft exhibits a high electropositivity attributed to the abundant presence of lysines and arginines on its surface. This elongated and highly positively charged cleft facilitates convenient access to electronegative HA chains (Jedrzejas et al. 2002). At the distal end of the cleft, a cluster comprising three negatively charged residues, Glu388, Asp398, and Thr400, forms a negative patch that is likely accountable for repelling the negatively charged products from exiting the cleft (product release). Furthermore, three closely placed aromatic residues, Trp291, Trp292 and Phe343 positioned between the positively charged center and the negative patch, form a small hydrophobic patch, which precisely anchors and positions the substrates for catalysis (Li et al. 2001). Specifically, Asn429, His479, and Tyr488 are recognized as the catalytic triad of spnHL; they interact with the chemical groups on substrates as well as other amino acid residues within enzyme (Nukui et al. 2003). Similarly to spnHL, mHyals from *S. agalactiae* (sagHL) exhibit an identical structural architecture and active site geometry. Compared with spnHL, sagHL maintains conserved catalytic residues including Asn429, His479 and Tyr488 while possessing an additional 82 residues at the N-terminal region that forms an extra β-sheet domain (β1-domain) (Fig. 2a). However, the presence of β1-domain does not exert any influence on protein function or activity; thus suggesting that this domain is not directly involved in binding and degrading of HA substrate (Mello et al. 2002).

### PL16 mHyals

Initially, PL16 family mHyals were exclusively found in streptococcal bacteriophage and prophage members. Pathogenic bacteria possess an outer HA capsule, which helps evasion from human immune system. Phages have coevolved with these bacteria-acquired HA-degrading enzymes to facilitate penetration of the outer shell during the infection process (Martinez-Fleites et al. 2009). In the genome sequence of *Streptococcus pyogenes*, three prophage sequences encoding for Hyal1, Hyal2 and Hyal3 were localized, and their corresponding crystal structures were determined (Martinez-Fleites et al. 2009; Mishra et al. 2009; Smith et al. 2005). Additionally, two other phage-encoded mHyals, HylP and SEQ2045, respectively originated from *S. pyogenes* phage H4489A and *Streptococcus equi* 4047 were also elucidated. Notably, a novel bacterial PL16 mHyal called Hyal_Sk from *Streptomyces koganeiensis* was reported for the first time in 2015 (Pavan et al. 2016). Similar to those from the PL8 family, PL16 mHyals also employed a β-elimination mechanism but exhibit HA-specificity and increased product promiscuity, resulting in a series of even O-HAs in final products (Table 2).

PL16 mHyals consist of three intertwoven polypeptides, forming a triple-stranded structure around the threefold crystallographic axis, resembling an irregular triangular hollow tube (Fig. 2d). Bacteriophage-derived PL16 mHyals fold into four domains: a mixed α/β-domain forming a globular capping at the N-terminal, an α-helical region with coiled coils, a central triple-stranded β-helix (TSβH) region composed of 16 anti-parallel β-strands, and a loosely packed α-helical bundle at the C-terminal (Mishra et al. 2009). The structure of Hyal_Sk shares similarities with truncated forms of HylP2 and HylP3, exhibiting solely the central β-helix (Messina et al. 2016). Due to the unavailability of enzyme-HA complex crystals, a precise characterization and description of the regions responsible for polysaccharide binding and associated structural alterations has not been achieved thus far. The catalytic center resides on the right-handed TSβH, which exhibits deeply concave shapes on three sides enriched with positively charged amino acids, providing an extended binding surface for long-chain carbohydrates. Asp137 and Tyr149 were proposed as the catalytic residues in HylP1, with Tyr acting as the catalytic base for proton abstraction from C5 of the GlcA and Asp involving in pKa maintenance (Smith et al. 2005). However, in HylP2 and HylP3, these catalytic dyads are not present. Tyr264 may participate in the catalytic activity of HylP2 by acting as an acid for hydrogen donation to the glycosidic oxygen (Mishra et al. 2009). Unfortunately, no crystal structure of phage Hyals complexed with HA substrate has been obtained yet. Therefore, further research is required to elucidate the actual catalytic residues and understand the process and mechanism of HA degradation by PL16 mHyals.

### Carbohydrate-binding modules

**Fig. 3:**
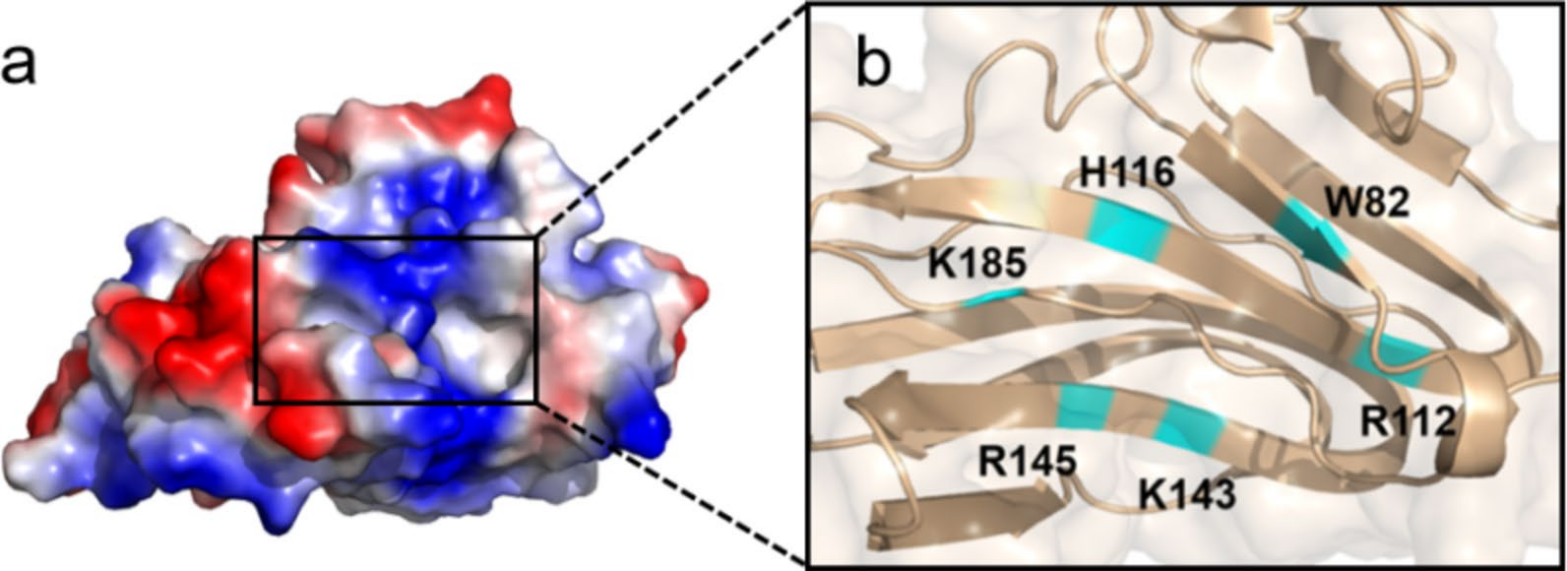
The tertiary structures of CBM70 (PDB entry 4D0Q). **a** The solvent-accessible surface of CBM70 is colored by electrostatic potential.** b** The magnified section shows the putative binding site of the CBM70 in the binding pocket

Carbohydrate-binding modules (CBM) are non-catalytic domains widely present in carbohydrate-active enzymes, serving as ancillary modules of parent enzymes for recognition and adhesion to carbohydrates (Shi et al. 2023). As of March 2024, a total of 101 CBM families have been identified and recorded in the CAZy database. HA-specific binding modules only exist in PL8 mHyals, and are categorized into the CBM70 family. The polypeptide consists of opposing anti-parallel β-sheets with five strands each, adopting a β-sandwich conformation with a jelly roll topology (Fig. 3). The sandwich structure exhibits subtle curvature, resulting in a channel that spans across the concave surface of the β-sheet and is presumed to be HA-bind site for its electrostatic properties with polysaccharides and conservation within the CBM70 family (Suits et al. 2014). Aromatic and polar amino acids located within the narrow cleft predominantly meditate intermolecular interactions between CBMs and their substrates (Cabral et al. 2024). The results of site-directed mutagenesis suggested that residues W82, R112, H116, K143, R145 and K185 situated in the biding groove are proved relevant to the recognition and combination of HA.

**Table 2:** Summary of characterized mHyals

Source	Name	Molecular mass	Activity	Optimum T/pH	Temperature stability, pH Stability	Action mode	Substrate spectrum	Activator	Inhibitor	Final products	Refs.
Bacteriophages											
*Streptococcus pyogenes* bacteriophage H4489A	HylP	39.5 kDa	–	37 °C/5.5	25–45 °C, > 70% 4.0–7.0, > 80%	Endo	HA	Mg^2+^, Ca^2+^	L-ascorbic acid, Zn^2+^, Cu^2+^, Ni^2+^, Co^2+^, SDS	∆HA4, ∆HA6	Baker et al. (2002) El-Safory et al. (2011) Singh et al. (2014)
*Streptococcus equi* 4047	SEQ2045	42 kDa	–	37 °C/7.0	44 °C, 20 min, 50%	Endo	HA	–	–	∆HA6, ∆HA8	Lindsay et al. (2009)
*Streptomyces koganeiensis* ATCC31394	Hyal_Sk	21.6 kDa	–	– /7.0	–	–	HA	–	–	∆HA4, ∆HA6, ∆HA8	Messina et al. (2016); Pavan et al. (2016)
*Streptococcus pyogenes* bacteriophage 10,403	HylP2	37 kDa	–	–/6.0	–	Endo	HA	–	Ascorbic acid	–	Mishra et al. (2006); Mishra et al. (2009)
Bacteria											
*Thermasporomyces composti* DSM22891	TcHly8C	90 kDa	10.91 U/mg^a^	70 °C/5.93	60 °C, 60 min, 90% 6.0–8.0, 12 h, > 50%	Exo	HA, CSC	Mg^2+^	SDS	∆HA2	Li et al. (2020)
*Thermasporomyces composti* DSM22891	TcHly8B	86 kDa	–	70 °C/6.6	20–60 °C, 1 h, > 80% 3.0–10.0, 12 h, > 80%	Exo	HA, CSA, CSC, CSD, DS	Na^+^, Ni^2+^	Li^+^, K^+^, Ba^2+^, Mg^2+^, Zn^2+^, Ca^2+^, Mn^2+^, Al^3+^, SDS	∆HA2	Wang et al. (2021a, b)
*Streptomyces coelicolor* A3 (2)	ScPL8Hyal	83 kDa	–	57 °C/5.2	–	Exo	HA, C4S, C6S	Mn^2+^, Ba^2+^, Ca^2+^	–	∆HA2	Elmabrouk et al. (2011)
*Citrobacter freundii* strain Cf1	HylC	90 kDa	1.62 × 10^4^ U/mg^b^	37 °C/5.5	50 °C, 1 h, > 50% 4.0–8.0, 2 h, > 60%	Endo	HA, CSA, CSC	Ca^2+^, K^+^, Zn^2+^, Ni^2+^, Li^+^	Mn^2+^, Al^3+^	∆HA2	Zhang et al. (2023)
*Vibrio alginolyticus* LWW-9	VaHly8A	83 kDa	223.65 U/mg^a^	30 °C/7.05	0–20 °C, 1 h, > 90% 5.0–10.6, 6 h, > 70%	Endo	–	–	Co^2+^, Ni^2+^, Zn^2+^, SDS	∆HA2	Wang et al. (2021a, b)
*Vibrio alginolyticus* LWW-9	VaHly8B	87 kDa	26.38 U/mg^a^	50 °C/7.05	0–30 °C, 1 h, > 90% 3.6–10.6, 6 h, > 70%	Endo	–	Mn^2+^, Co^2+^, Ni^2+^	SDS	∆HA2	Wang et al. (2021a, b)
*Yersinia* sp. 298	YsHyl8A	115.4 kDa	11.19 U/mg^a^	40 °C/7.5	30 °C, 1 h, > 50% 6.0–11.0, 6 h, > 80%	Endo	HA, CSA, CSB, CSD	Ca^2+^, Mg^2+^, Na^+^	Ba^2+^, Zn^2+^, Mn^2+^, Ag^+^, Ni^+^, SDS, EDTA	∆HA2	Zhang et al. (2022a, b, c)
*Clostridium perfringens*	–	96 kDa	–	42 °C/5.7–6.2	30–50 °C, 30 min, > 50%	–	HA, CSA, CSB, CSC	–	Pb^2+^, Zn^2+^, Cu^2+^, Al^3+^, EDTA	–	Zukaite et al. (2000)
*Bacillus sp.* CQMU-D	HAaseD	123 kDa	–	40 °C/7.0	20–50 °C, 1 h, > 80% 7.0–10.0, 2 h, > 60%	Endo	HA, CSA, CSB, CSC	Ca^2+^, Mg^2+^, Ni^2+^	Ba^2+^, EDTA, SDS	∆HA2	Wang et al. (2023)
*Microbacterium* sp. H14	HCLaseM	85.9 kDa	278.3 U/mg^a^	35 °C/7.0	40 °C, 1 h, 50% 6.0–8.0, 12 h, 90%	Endo	HA, CSA, CSB, CSC, CSD	Al^3+^	Hg^2+^, Fe^2+^, Cu^2+^	∆HA2	Sun et al. (2019)
*Vibrio* sp. H240	HCLaseV	90 kDa	6.092 U/mg^a^	40 °C/7.0	30 °C, 1 h, > 80% 6.0–8.0, 12 h, > 80%	Endo	HA, CSA, CSB, CSC	Li^+^, Ba^2+^, Mg^2+^	Ba^2+^, Zn^2+^, Ca^2+^, Cu^2+^, Ni^+^, Fe^3+^, SDS, EDTA	∆HA2	Wang et al. (2022)
*Vibrio* sp. FC509	HCLase	90 kDa	4.5 × 10^5^ U/mg^a^	30 °C/8.0	0–40 °C, 24 h, > 50%	–	HA, CSA, CSC, CSD, CSE	Li^+^, Na^+^, K^+^	Ag^+^, Hg^2+^, Pb^2+^, Ni^2+^, Fe^2+^, Cu^2+^, Zn^2+^, Fe^3+^, Cr^3+^	–	Han et al. (2014)
Fungi											
*Fistulina hepatica* DSM4987	FHHAase	150 kDa	–	20 °C/4.0	–	Endo	–	–	–	∆HA4, ∆HA6, ∆HA8, ∆HA10	Bobkova et al. (2018)
*Talaromyces stipitatus* DSM2425	TSHAase	30 kDa	–	43 °C/3.0	60 °C, 5 h, > 60%	Endo	HA	–	–	HA2	Bobkova et al. (2018)
*Penicillium purpurogenum*	HAase-PP	31.7 kDa	–	43 °C/3.0	43 °C, 10 d, > 95% 50 °C, 1 h, > 80%	Endo	HA	–	–	HA2	Bakke et al. (2011)
*Pseudozyma aphidis*	–	130 kDa	–	37–45 °C/3.0	18–37 °C, 1 h, > 95%	Endo	HA	–	–	–	Smirnou et al. (2015)
*Cryptococcus laurentii*	–	–	–	37 °C/6.0	45 °C, 1 h, 20%	Endo	HA	–	–	–	Smirnou et al. (2015)

^a^One unit was defined as the amount of the enzyme that release 1 μmol unsaturated oligomers per minute detected by absorbance at 232 nm

^b^One unit was defined as the amount of the enzyme that release 1 μg of reducing sugar per hour detected by DNS method

## Enzymatic properties of mHyals

mHyals derived from various organisms exhibit diversity in enzymatic properties and characteristics. These catalytic properties, including optimum temperature, optimum pH, thermal stability, pH stability and substrate spectrum, tremendously influence the degradation process of the substrate and determine the qualities and quantities of products obtained. A summary of the enzymatic properties of characterized mHyals is presented in Table 1.

### Influence of calcium ions on mHyals

As early as 2000, it was reported that Ca^2+^ activates mHyals. David et al. discovered an 11-fold activation of group B *streptococcus* (GBS) mHyal in the presence of 8 mM Ca^2+^ (Pritchard et al. 2000). Atsushi et al. discovered that mHyals BniHL exhibited maximum activity in degrading HA, chondroitin sulfate A (CSA), and chondroitin sulfate C (CSC) when exposed to Ca^2+^ concentrations of 2 mM, 50 mM, and 100 mM respectively. In addition, Ca^2+^ positively effects the thermal stability of BniHL, while decreasing its pH stability (Kurata et al. 2015). However, it should be noted that the presence of Ca^2+^ is not essential for mHyal activities, although Ca^2+^ has been confirmed to be essential for some other GAG lyases. For example, two Ca^2+^-binding sites are identified in heparinase I and the interaction between calcium and heparinase I helps the enzymes to function properly (Liu et al. 1999; Zhou et al. 2023). Pectate lyase contains three calcium binding sites, which have clearly defined functions in the binding substrate and promote catalysis (Zheng et al. 2021). Most chondroitin AC lyases also exhibit high activity dependence on calcium ions (Fan et al. 2022).

Among phage mHyals,a collagen-like Gly-X–Y motif was only observed within the N-terminal region of HylP and SEQ2045, and proved to be functional in the enzyme regulation through interaction with calcium ions (Singh et al. 2014). However, the precise mechanism by which Ca^2+^ influences the activity of mHyals from other families remains unknown at present. Crystal structures suggest that calcium ions might modulate the enzyme activity through regulation of C-terminal β-domain (Li et al. 2000). In the presence of Ca^2+^, conformation changes in loops, probably originating from the β-domain could open up the substrate-binding cleft and facilitate catalysis (Jedrzejas 2000). As previously mentioned, neutralization of negatively charged uronic acid derived from the HA is critical for degradation. The interactions between Ca^2+^ and glucuronate group could stabilize the enol anion intermediate by resonance and provide another plausible explanation for the Ca^2+^ coordination in GAG lyases (Yang et al. 2020). Additionally, structural studies on HA indicated that the calcium cation can simultaneously bind to the polymer chains that carry negative charges and weaken the intramolecular hydrogen-bond network of HA (Giubertoni et al. 2021), thereby increasing the flexibility of the HA chain and facilitating interaction between Hyals and its substrate. Further structural investigations are imperative to comprehensively elucidate the precise role of calcium ions in the degradation of polysaccharides.

### Temperature properties of mHyals

The optimal temperature of most mHyals ranges between 37 and 45 °C. However, certain mHyals with exceptional catalytic activities at either high or low temperatures have also been identified in nature. For example, a mHyal discovered in *Talaromyces stipitatus* exhibits an optimal temperature of 20 °C and demonstrates less than half of its activity at 40 °C. Although the occurrence of mHyal with low-temperature activity has been scarcely reported thus far, they have been found exclusively in micromycetes. By contrast, a few mHyals with optimal temperatures exceeding 50 °C have been heeded (Elmabrouk et al. 2011; Li et al. 2020; Wang et al. 2021a, b; Wang et al. 2021a, b). Two thermophilic mHyals from *Thermasporomyces composti* DSM22891, TcHly8B and TcHly8C, have an optimum temperature of 70 °C, and remain at least 80% activity after exposure to a temperature of 60 °C for 1 h, which are the most thermostable mHyals documented to date (Li et al. 2020; Wang et al. 2021a, b). The thermostability of proteins is influenced by various factors, including the presence of salt bridges and hydrogen bond networks, hydrophobic interactions, cavity sizes, conformational changes, tendencies towards secondary structural elements, as well as flexibility and rigidity (Hong et al. 2024; Kumar et al. 2023). The authors predited the structures of TcHly8B and TcHly8C, and conducted an analysis of the salt bridges. The findings revealed a great abundance of salt bridges, which may serve as a crucial determinant for their exceptional thermal stability. Thermostable mHyals possess numerous advantages, including enhanced solubility of polysaccharide substrates, increased degradation rate, and reduced potential for microbial contamination growth (Zheng et al. 2019). Nevertheless, there remains a dearth of studies employing protein engineering strategies to improve thermostability of mHyals. However, chemical modification techniques utilizing macromolecules like polyethylene glycol (PEG) and poly (amide amine) have proven effective in improving enzyme thermal stability of mammalian Hyals (Amin et al. 2021; Soozanipour et al. 2022), which also can be adapted in the research on bacterial Hyals engineering.

### pH properties of mHyals

The majority of mHyals are neutral or slightly acidic enzymes and are found to exhibit optimal activity at a pH range of 5.5–7.0. Certain mHyals can retain their activity even after incubation in neutral pH buffer systems, while some demonstrate resistance to acidic or alkaline conditions. An alkalophilic GAG lyase YsHyl8A, which showed highly HA-degrading activity, was discovered from marine bacteria *Yersinia sp.* 298. YsHyl8A has an optimal activity at pH 7.5 and remains stable for up to 6 h within the pH range of 6.0–11.0, retaining more than 80% of its initial activity (Zhang et al. 2022a, b, c). Similarly, the thermotolerant mHyal TcHly8B also demonstrates remarkable pH stability by retaining over 80% enzyme activity after being incubated in a wide pH range of 3.0–10.6 for a duration of 12 h (Wang et al. 2021a, b). However, most fungal mHyals display an acidic optimal pH, ranging from 3.0 to 4.0. For example, the first Hyal derived from filamentous fungi, FhHAase, has an optimal pH at 4.0, and remains activity even after an 8-day incubation under optimum reaction conditions (pH 4.0 and 20 °C) (Bobkova et al. 2018). Additionally, mHyals detected in *Talaromyces stipitatus* (Bobkova et al. 2018), *Penicillium purpurogenum* (Bakke et al. 2011) and *Pseudozyma aphidis* (Smirnou et al. 2015) have optimal pH and temperature around 3.0 and 43 °C respectively along with decent thermostability properties. It is worth noting that fungi serve as significant sources for acidic enzymes due to their adaptation towards growth environments characterized by acidity (Hassan et al. 2019).

### Activators and inhibitors

Besides calcium ions, other metal ions and chemical reagents also exhibit differential modulation on the activities of mHyals. L-ascorbic acid (Vitamin C, Vc) has been identified as a competitive inhibitor of spnHL and sagHL, with IC_50_ values of 34.8 mM and 6.1 mM respectively (Botzki et al. 2004). The structure of SpnHL-Vc complex elucidated that Vc establishes 25 interactions with active sites of the enzyme, wherein hydrophobic interactions with Trp292 and Tyr408 exert a dominant influence (Li et al. 2001). Moreover, it has also been reported that Vc exhibits inhibitory effects on PL16 mHyals. The molecule was speculated to occupy the saccharide binding groove in each face of the triangular tube on HylP2, with IC_50_ values of 1 mM being detected (Mishra et al. 2009). However, unlike HylP2, HylP is inhibited by Vc in a non-competitive manner. In-vitro enzyme inhibition experiments revealed that the *K*_m_ value of HylP remained constant in both the absence and presence of varying concentrations of L-ascorbate. Additionally, a Lineweavere-Burke plot (double reciprocal) intersected at the abscissa axis, indicating non-competitive inhibition by Vc (Singh et al. 2014). Interestingly, SEQ2045 showed no response to Vc even at concentrations up to 20 mM (Lindsay et al. 2009), confirming the diverse properties of HLs.

The effects of metal ions vary significantly among different species’ mHyals. Mn^2+^ and Ni^2+^ were found to enhance the enzymatic activity of VaHly8A (Wang et al. 2021a, b), HAase-*B* (Guo et al. 2014) and BniHL (Kurata et al. 2015), while strongly inhibiting that of YsHyl8A (Zhang et al. 2022a, b, c). Mg^2+^ acts as an activator in most investigated mHyals, but exhibits apparent inhibition of TcHly8B (Wang et al. 2021a, b). The impact of Na^+^ concentration has been explored to characterize the salt stability of enzymes. mHyal from *Clostridium perfringens* displayed maximum activity at a NaCl concentration equal to 0.1 M (Zukaite et al. 2000); whereas mHyal from *Brevibacterium halotolerans* maintained high activity up to a NaCl concentration of 2 M, representing its halotolerant nature and salt stability (Patil et al. 2021). The effects of metal cations on enzymes can arise from either substrate binding with direct participation of metal ions or alterations in enzyme conformation or surface charge disturbance (Lin et al. 2022; Zeng et al. 2014). EDTA, a metal ion chelator, modestly decreases the activity of most mHyals by 10%–30%, without complete inhibition. Conversely, SDS, an ionic detergent capable of significantly altering protein tertiary structure, completely inhibits mHyals at any concentration. Notably, EDTA has an inconspicuous impact on HAase-PP (Bakke et al. 2011), a fungi enzyme belonging to GH16 family; similarly, marine GAG lyase YsHyl8 shows considerable resistance against SDS (Zhang et al. 2022a, b, c).

### Substrate spectrum

CS and HA are optical isomers, which only differ in stereoisomerism of the C4 on the repeating N-acetylhexosamine moiety (Tao et al. 2017). Typically, PL8 mHyals have a preference for HA as their primary substrate, with partial activity towards CS; however, they also demonstrate variability in their substrate scope. TcHly8C displayed limited activity toward CSC but lacked any activity toward other sulfated CSs, including CSA, chondroitin sulfate B (CSB) and chondroitin sulfate D (CSD) (Li et al. 2020). Conversely, TcHly8B displayed a fraction of activity towards CSA, CSC, CSD and even dermatan sulfate (DS) (10–20% of its activity towards HA) (Wang et al. 2021a, b). Importantly, certain GAG lyases exhibit comparable enzymatic activity towards both HA and CS variants, which is different from the conventional PL8 mHyals (Han et al. 2014; Lin et al. 2022; Wang et al. 2022). The absence of the Phe residue in the aromatic patch of HCLaseM eliminates structural conflicts between the enzyme and 4-sulfated CS, thereby potentially facilitating the degradation of all kinds of sulfated CSs (Sun et al. 2019). On the contrary, reported mHyals from PL16 and GH16 families have been reported to show exclusive specific activity for HA. At comparable protein concentrations, the phage enzyme HylP demonstrated a 30-fold higher rate of HA cleavage compared to the *Streptomyces* mHyal (Baker et al. 2002). Hence, it can be inferred that PL16 and GH16 mHyals could serve as excellent choices for rapid and selective cleavage of HA in specific usage scenarios.

## Biological functions of mHyals

### mHyal in invasion to hosts

**Fig. 4:**
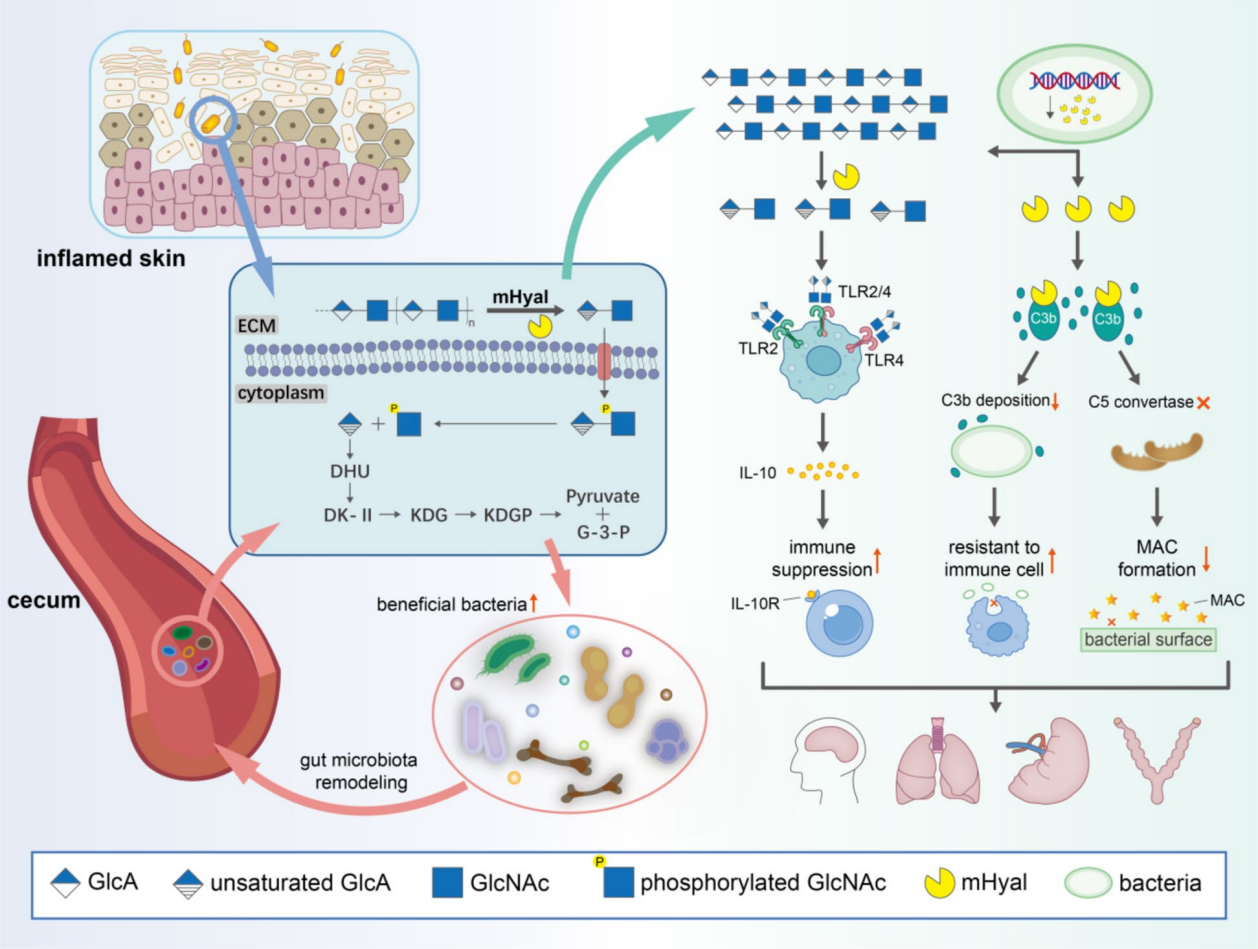
Biological functions of mHyals

mHyals can be produced as virulence factors in various pathogenic bacteria, and contribute to their pathogenicity and infectivity (Dokoshi et al. 2020; Hynes et al. 2000). *S. agalactiae*, also known as GBS, is a Gram-positive pathogen responsible for invasive infections in humans, inducing neonatal pneumonia (Gessner et al. 2005) and meningitis (Gravett et al. 1996). Clinic studies have revealed that GBS strains producing higher levels of extracellular mHyals exhibit greater virulence compared to those with lower production (Rolland et al. 1999). The involvement of mHyals in host invasion encompasses multiple mechanisms. Primarily, mHyals disrupt the biophysical barrier of host connective tissues while decreasing the viscosity and density of the ECM, thereby facilitating invasion and dissemination within the host (Zamboni et al. 2023). Furthermore, the presence of polysaccharide utilization loci for HA (PUL_HA_) renders the bacteria's ability to utilize HA as a nutrient source for survival and colonization (Feng et al. 2022; Hirayama et al. 2009). Complete degradation and utilization models of HA have been proposed in certain bacterial species (Oiki et al. 2019a, b; Oiki et al. 2019a, b). Initially, extracellular and cell-surface mHyals degrade HA into ∆HA2, which is subsequently imported into the cytoplasm through a transporter system for phosphorylation reactions. The imported ∆HA2P is then degraded to constituent monosaccharides by unsaturated glucuronyl hydrolase. Non-enzymatic conversion of unsaturated uronates leads to the formation of 4-deoxy-L-threo-5-hexosulose-uronate (DHU), which is ultimately metabolized to pyruvate and glyceraldehyde-3-phosphate (G-3-P) through consecutive reactions involving isomerase, dehydrogenase, kinase, and aldolase (Fig. 4). Interestingly, two genes encoding mHyals were identified within the PUL_HA_ of *Vibrio alginolyticus* strain LWW-9. These two mHyals exhibit distinct biochemical properties, which provides evidence for bacterial adaption to variable environmental conditions (Wang et al. 2021a, b).

### mHyal in disease/infection development

mHyals also participate in regulating the host’s immune response. Wang et al. reported that the expression of the *hylB* gene, encoding mHyal, was associated with intracellular survival of GBS and proinflammatory cytokine expression. In the absence of mHyals, the isogenic *hylB* mutant exhibited declined survival in macrophages compared to the wild type, accompanied by a notable increase in the release of proinflammatory cytokines. These findings suggest that mHyals are beneficial for the survival and function of GBS (Wang et al. 2014). In a mouse acute lung injury model, ∆HA2 produced by GBS can attenuate inflammatory levels to evade immune detection (Kolar et al. 2015). Moreover, hylB enables GBS to induce immune suppression in a TLR2/4- and interleukin-10 (IL-10) -dependent manner, leading to increased rates of ascending infection and preterm birth (Coleman et al. 2023; Vornhagen et al. 2016). mHyals have also been observed in human commensal *Cutibacterium acnes*, which is strongly associated with skin health (Hajam et al. 2023). *C. acnes* strains express two types of mHyals, HylA and HylB, which show distinct properties and remarkable clinical correlation with acne or health. HylA induces a strong TLR2-dependent inflammatory response by breaking down HA into larger fragments, while HylB exclusively degrades HA into ∆HA2, resulting in reduced inflammation. The functional divergence between HylA and HylB arises from subtle structural differences in substrate binding cleft, and serves as a major determinant behind health status and acne phenotype associated with *C. acnes*.

Moreover, in a recent study, HylS’, a truncated fragment of *Streptococcus suis*’s mHyal, has been reported to contribute to immune evasion by interacting with the host complement factor C3b (Xu et al. 2024). HylS inhibits the deposition of C3b, thereby impacting bacterial survival within the host and blocking the synthesis of C5 convertase. Ultimately, this leads to a reduction in membrane attack complex (MAC) formation on the bacterial surface. Bacteria lacking deposited C3b on the surface show resistance against immune cells, facilitating further invasion into host tissue and organs.

### mHyal in intestinal flora regulation

mHyals in intestinal microbiota may be associated with the absorption and metabolism of HA (Zheng et al. 2023). Experimental findings made out that HA could be depolymerized into O-HAs by the cecal content of rats, whereas this degradation did not occur when treated with artificial gastric juice and intestinal juice (Kimura et al. 2016). It has been reported that certain species of *Bacteroides* from the human gut microbiota and *Enterococcus faecalis* from human feces can degrade HA (Kawai et al. 2018; Ndeh et al. 2020). Under the action of microorganisms, HA was degraded into O-HAs, all of which could be utilized while simultaneously inducing modifications to the composition of gut microbiota (Pan et al. 2021). The abundance of short-chain fatty acids-producing bacteria, such as *Bifidobacteria* spp., *Dialister* spp. and *Faecalibacterium* spp. were increased and further influenced the level of metabolites, which demonstrates a positive impact on intestinal regulation (Zhao et al. 2023). Shi et al. evidenced that the therapeutic effect of HA treatment on bacterial colitis was associated with increased clearance of *Citrobacter rodentium* and alleviation of pathogen-induced gut dysbiosis (Mao et al. 2021), providing news perspectives on the potential application of mHyals by reshaping the composition of intestinal microbiota.

## Function-inspired biotechnological applications of mHyals

### Applications in diagnosis and treatment of bacteria

#### Reporter molecules for detection of bacterial infections

In recent years, the detection of bacteria-based mHyal responsiveness has undergone a revolutionary transformation and has found extensive applications in various fields, including sensing and other biomedical domains (Krismastuti et al. 2015). The HA polymer network is enzymatically cleaved by mHyals, generating GlcA fragments. Interestingly, a significant upsurge in mHyals is observed just prior to the initiation of the exponential growth phase. This enzymatic degradation process has been harnessed for developing transducers that enable efficient detection and evaluation of bacterial virulence (Mohammed et al. 2022).

In this regard, Tücking et al. (2018) developed thermally hydrocarbonized porous silicon films modified with HA for on-site detection of *S. aureus*. The proposed approach entails impregnating a crosslinked HA methacrylate/polyethylene glycol diacrylate hydrogels into the porous silicon electrode. When reacting on the inner walls of the channels, mHyals blocked the electroactive ion difussion, resulting in a reduction in the voltammetric signal. Differential pulse voltammetry was employed to quantify the decrease in current resulting from increased electrostatic repulsion encountered by the external redox probe. The concept of electrochemical detection for sensing bacterial infection was validated by successfully detecting enzymatic degradation in various media, including buffer, bacterial supernatants, and artificial wound fluid.

Moreover, Alfredo et al. (de la Escosura-Muñiz et al. 2019) utilized nanoporous alumina membranes of mHyals for the electrical evaluation of bacterial pathogens. The proposed approach relied on the electrical monitoring of nanochannels that are obstructed during mHyals immunorecognition. By taking indium tin oxide/poly (ethylene terephthalate) (ITO/PET) as electrodes and capitalizing on the noble surface properties of ITO/PET in conjunction with the biofilm-resistant characteristics of nanoporous alumina membrane, bacteria can be cultured and secreted enzymes can be captured within the nanochannels. Upon the interaction between antibody and mHyals within the nanochannels, an immunocomplex was formed, causing a partial hindrance to the dispersion of electroactive species ([Fe(CN)_6_]^4−^) across the membrane towards the electrode. Consequently, this led to a reduction in voltammetric signal during oxidation. This highly sensitive method exhibits an impressive detection limit of 64 UI/mL (17.3 U/mg) mHyal, providing a time-efficient and operationally convenient means for monitoring bacterial virulence/invasion.

#### Penetrate biofilms for anti-bacterial therapies

Bacterial biofilm, a naturally developing matrix of microbial communities, is formed through bacterial attachment, colonization and expansion, which can adhere to multifarious surfaces with abiotic devices no exception (Dsouza et al. 2024; Sauer et al. 2022). This invasion typically evades the human immune system and shows resistance to antimicrobial agents, posing a grievous threat to human health (Suresh et al. 2019). The biofilm matrix primarily consists of DNA, proteins and extracellular polysaccharides. Disrupting these matrix components can be a viable approach to disperse and manage established bacterial biofilms (Kanwar et al. 2019). The expression of mHyal contributes to the prevention of *S. aureus* biofilm formation and disruption of established biofilm (Patel et al. 2021), and could impact the formation of biofilm-like synovial fluid aggregate in joint infection (Ibberson et al. 2016). mHyal is also effective in the detachment and spread of *Streptococcus intermedius* from biofilm, which may be significant in the pathogenicity of this micro-organism (Ibberson et al. 2016). Additionally, bacteriophages have evolved a wide diversity of depolymerases, which could potentially enhance phage infection by degrading polymeric substances in bacterial surface and biofilm communities (Wang et al. 2024). Phage-encoding Hyals have been found in multiple pathogens, such as the group A S*treptococcal* phage phiNIH1 and *S. equi* phage P9 (Pires et al. 2016). These phages containing HA-degrading enzymes could penetrate HA capsules, and are thought to be a promising strategy to combat multidrug-resistant pathogens against bacterial infection (Nobrega et al. 2018).

### Applications in biomedicine engineering

#### Multiple functions of mHyals for cancer therapies

HA is abundant in the ECM and is overexpressed in various malignant tumors, creating a biophysical barrier that hinders immune cell infiltration and limits the delivery of anticancer agent (Auvinen et al. 2014; Jacobetz et al. 2013). Therefore, degrading excessive tumor-derived HA has been proposed as a strategy to improve drug delivery efficiency. Studies have evidenced that mHyals process practical anti-cancer activities and hold promise for future therapeutic and pharmaceutical applications. For instance, a mHyal originated from marine *S. aureus* exhibited potent antioxidant properties and displayed in-vitro anti-breast cancer effects. This enzyme demonstrated significant efficacy in suppressing the growth of MCF-7 breast cancer cells, resulting in complete cellular demise when administered at a concentration of 80 µg/mL. Additionally, it generated free radicals that inflicted substantial harm upon cancer cells, comparable to the effects of the standard drug paclitaxel (Thirumurthy et al. 2023). Another example is bacteriophage Hyal HylP, which was reported to significantly suppress HA-mediated breast carcinoma growth and progression. Unlike saturated HAs metabolized by HA hydrolase that stimulate the growth of breast carcinoma cells through Erk1/2 kinase activation, unsaturated O-HAs produced by HylP efficaciously downregulated this signaling pathway, thereby attenuating proliferation, migration and invasion of breast cancer cells (Lee et al. 2010).

At present, candidate drugs targeting HA have exhibited promising potential in enhancing the permeability of chemotherapeutic agents. However, the occurrence of severe negative consequences linked to systemic depletion of ECM in peripheral healthy tissues restricts their utilization at higher and more efficacious doses (He et al. 2020a, b). Considering this limitation, engineered bacteria that specifically target tumors have been developed and garnered wide attention as a promising anti-cancer therapy. Ebelt et al. reported an attenuated *Salmonella typhimurium* strain expressing functional mHyal (bHs-ST), which could target tumor-associated HA in pancreatic ductal adenocarcinoma and colonies within orthotopic tumors following systemic administration. Subsequently, the induced expression of mHyal within the tumor microenvironment leads to depletion of tumor-derived HA and in turn prominently enhances diffusion capabilities for *S. typhimurium* within desmoplastic tumors (Ebelt et al. 2020). The utilization of bHs-ST demonstrates a promising and innovative strategy to target the tumor ECM, potentially minimizing toxicity in off-tumor areas while maximizing drug delivery into highly desmoplastic tumors, thus providing a novel therapeutic option for cancer treatment. Moreover, an engineered *E.coli* Nissle capable of distributing two different recombinant proteins—Cytolysin A and mHyal—via membrane vesicles can remodel the tumor stroma, synergistically enhancing penetrability of anticancer agents and inducing tumor resolution (Thomas et al. 2022).

#### Trigger feature of mHyals for HA-responsive drug delivery

Antibiotic resistance is a growingly severe problem due to the indiscriminate use of antibiotics (Mohammed et al. 2022). Excitingly, Hyal-responsive drug delivery systems were developed to combat this challenge. This delivery system usually includes traditional antibiotics, such as clarithromycin and gentamicin, with a HA upper layer (Zafar et al. 2023; Zhang et al. 2022a, b, c). Hyal is known to be expressed and secreted by pathogenic bacteria associated with wound infections, particularly in gram-positive bacteria, such as *Streptococcus*, *Staphylococcus*, *Streptomyces*, and *Clostridium* (Hynes et al. 2000). In the presence of enzyme-producing bacteria, HA can act as a capping agent in response to mHyal presented at the infection microenvironment, releasing coated formulation (Shen et al. 2016).

A nanocarrier containing HA/AgNPs/gentamicin (HB/Ag/g) was developed by Yu et al. (2020) demonstrating an excellent bacteria disinfection capability through a synergistic effect. mHyals led to the specific rupture of the HB/Ag/g nanocarrier, triggering the release of AgNPs and gentamicin, and performing robust antibacterial function through different mechanisms. Moreover, an HB/Ag/g coating was attached to the polydopamine-modified chitin hydrogel (CPH), as a wound dressing named HB/Ag/g@CPH, not only effectively inhibits bacterial growth and adhesion, but also having no effect on cell attachment and proliferation. A similar scheme can also apply to the development of antibacterial medical devices. Sutrisno et al. fabricated an effective implant with antibacterial properties for orthopedic implantation (Sutrisno et al. 2018). The titanium nanotube (TNT) loaded with bone morphogenetic protein 2 (BMP2) on the surface was modified with chitosan (Chi)/sodium hyaluronate-lauric acid (SL) via spin-assisted layer-by-layer technique, termed TNT/BMP2/(Chi/SL/Chi/Gel)_4_. mHyals could induce the liberation of lauric acid from the SL multilayer and expedite the discharge of BMP2 in the system. Due to the damage of lauric acid on bacteria and the induction of BMP2 on osteoblast differentiation, this implant demonstrates excellent antibacterial capability and biocompatibility in in vitro usage. This novel mHyal-responsive, on-site and on-demand drug delivery system allows therapeutic release only when mHyal-secreting strains are present, thereby ameliorating the formation of bacterial resistance (Bean et al. 2014; Wu et al. 2019).

### Disintegrate HA coat for DNA transformation enhancement

Certain microorganisms, such as group A *Streptococcus* (Wilde et al. 2023), synthesize HA-rich capsules to provide protection against external damage. However, these viscous polysaccharide layers also impede targeted substance transfer in biochemical research. Genetic transformation poses a challenge for bacteria with HA-rich capsules. Abdelhak proposed an alternative method for the genetic transformation of *Pasteurella multocida* X73 by co-cultivating it with *S. aureus*, resulting in a satisfactory transformation rate, which greatly facilitated DNA transfer in encapsulated bacteria (Abdelhak 2009).

Recombinant proteins and peptides have been commonly used as traditional subunit vaccines, but now plasmid DNA-based cancer vaccines are being recognized as feasible alternatives. Nevertheless, their limited potency remains a significant challenge in DNA vaccination (Arbyn et al. 2011; Lopes et al. 2019). Preclinical studies utilizing Gene Electro-Transfer (GET) have demonstrated the positive effects of mHyals, resulting in increased transfected cells and enhanced expression of encoded genes (Peri et al. 2020). To address concerns regarding low purity, variable potency, and uncertain safety associated with animal-derived Hyals, Robertis et al. employed rHyal-sk to optimize intramuscular GET-based protocols in mice (De Robertis et al. 2018). The results showcased the safety and efficacy of rHyal-sk when administrated intramuscularly alongside GET, demonstrating no signs of toxicity, proficient plasmid intake capacity, favorable activation of inflammatory responses, and minimal immunogenicity. The incorporation of microbial rHyal-sk as a novel component within immunotherapy protocols involving plasmid delivery can augment DNA electrotransfer for pioneering therapeutic approaches against cancer.

### Production and processing of HA

Fertile research has evidenced a close correlation between the biological function of HA and its molecular weight (Qiu et al. 2021). High-molecular-mass HA (HMW-HAs) exhibit exceptional hydratability, viscoelasticity and biocompatibility, rendering them valuable fillers and lubricants in tissue engineering and clinical medicine (Chen et al. 2016; Jin et al. 2020). On the other hand, LMW-HAs play pivotal roles in the anti-inflammatory response and skin wound healing (King et al. 2020; Olsson et al. 2018). Compared to HMW-HAs, LMW-HAs possess a greater propensity to penetrate the skin efficiently improving the moisturizing properties of cosmetic and daily chemical products (Jin et al. 2020). Furthermore, smaller molecular weight HA, O-HAs, significantly contribute to angiogenesis, fibroblast proliferation as well as suppression of tumor growth (Kim et al. 2018; Quartey et al. 2024; Yao et al. 2021). Physical, chemical and biological techniques are the predominant and effective approaches for modulating the molecular weight of HA (Table 3). Physical degradation encompasses thermal treatment, mechanical manipulation and radiation exposure. Chemical approaches refer to depolymerize specimens with acid/alkaline solutions or oxidants, such as O_3_, H_2_O_2_ and NaClO. Both physical and chemical methods typically necessitate prolonged processing time while posing challenges in achieving complete degradation (Jabbari et al. 2023). Biological techniques employ Hyals as enzymatic scissors to depolymerize HA under neutral operating conditions, which have been developed into effective tools for regulating HA fragment size and O-HAs production (He et al. 2020a, b; Pang et al. 2022).

**Table 3:** Degradation methods of HA

Method		Strategy	Molecular weight or type of HA	Product distribution	Refs.
Physical degradation	Ultrasonic treatment	200 mg of HA is solubilized in 20 mL of distilled water (0.15 M NaCl). Ultrasonic irradiation (50 W, 25 kHz) was applied at 4 °C for a sonication period lasting 4 h	181 kDa	1.48	Hafsa et al. (2017)
	Gamma ray irradiation	HA powder was irradiated by a cobalt-60 irradiator at an absorbed dose of 50 kGy (11.1 PBq, 10 kGy/h) at 22 ± 0.5 °C	211 kDa	2.27	Choi et al. (2010)
	Thermal treatment	HA powder was heated in a dry oven for 52 h at 90 °C	229 kDa	2.39	Choi et al. (2010)
Chemical degradation	Acid depolymerization	Acid hydrolysis was performed in duplicate after addition of 5 mg H_3_PO_4_ per mg HA (pH = 1.71) to 10 mL of 1 g/L HA solutions (dissolved in ultrapure water) at 40 °C for 24 h	397.3 Da (HA2)	–	Valcarcel et al. (2020)
	Ozone treatment	500 ml 1% HA solutions (dissolved in 0.15 M NaCl) was treated with ozone under the flow rate of 46 ± 5 mg/min for 120 min at 40 °C	87 kDa	1.66	Yue (2012)
Biological methods	mHyal from *Streptococcus pyogenes* bacteriophage H4489A	5 mg/ml HA in acetate buffer was mixed with 500 μl mHyal solution and incubated at 37 ◦C for 3 h	Unsaturated O-HA (1–20 mers)	–	El-Safory et al. (2010)
	mHyal from *Streptococcus*	240 kDa HA was incompletely digested by *Streptococcus* Hyal to generate a heterogeneous mixture of HA fragments	10 kDa HA fragments mixture	–	Tolg et al. (2014)
	mHyal from *Streptomyces*	Enzymatic HA hydrolysate O-HAs were digested by hyal from *Streptomyces*	∆HA2	–	Han et al. (2022)
	In situ degradation	mHyal secreted by *S. zooepidemicus* itself could degrade produced HA in the fermentation broth	125 kDa	–	Mei et al. (2019)

Commercially available bovine testicular Hyal (Kakizaki et al. 2010) and leech Hyal (Lv et al. 2016) have been utilized for preparation of saturated O-HAs, while mHyals have been employed for the production of unsaturated O-HAs. El-Safory et al. produced unsaturated HA oligomers (1–20 mers) with recombinant mHyal of *S. pyogenes* bacteriophage H4489A, and found that the mixture shows stronger antioxidant activities compared to natural HA and recombinant mHyal (El-Safory et al. 2010; El-Safory and Lee 2010). A heterogeneous mixture of 10 kDa HA fragments was generated by *Streptococcus* Hyal and was evidenced by the simulation for fibroblast migration in scratch wound assays at a concentration of 10 μg/ml (Tolg et al. 2014). Moreover, different sizes and structures of O-HA and its derivatives have distinct pro- and anti-inflammatory properties. Saturated HA4 promotes inflammation, whereas HA2 and ∆HA2 could inhibit inflammation, respectively. The enzymatic ∆HA2 is beneficial against lipopolysaccharide (LPS)-induced immune responses, highlighting the importance of acetyl group presence and configuration in such an effect (Han et al. 2022). Additionally, certain HA-yielding strains also could produce mHyal, leading to a decrease in HA molecular weight during the fermentation process as cultivation time prolongs (Huang et al. 2008). After centrifugation to remove the cells from *Streptococcus zooepidemicus* fermentation broth, Mei et al. allowed the solution to stand for 72 h. During this period, facilitated by supernatant mHyals, a significant decrease in the molecular weight of HA occurred from 1890 to 125 kDa; thus providing a simple method for in-site depolymerization-based production of LMW-HAs (Mei et al. 2019).

### Versatile functions of HA-specific CBMs

CMB70, an HA-binding module, exhibits the ability to discriminate polysaccharides with subtle structural differences and demonstrates a strong affinity toward HA (Suits et al. 2014). Building upon this interaction, Mei et al. pioneeringly established a lateral flow immunoassay (LFIA) method for the identification of HA by incorporating SrCBM70 from *Streptococcus ruminantium* sp. into LFIA. When detecting liquid without HA, both the C line and T line were observed. The mobility of CBM gradually decreased in correlation with the increase in HA concentration within the samples. Consequently, as the concentration of HA increased, the intensity of the T line progressively diminished and was completely suppressed at a HA concentration of 1.5 μg/mL. This method could complete the assay within 5 min with 0.1 μg/mL detection limit, providing a convenient and efficient solution for discernment of HA-containing products (Mei et al. 2022).

CBMs are also applied to modification for polysaccharide-based materials, such as cellulose fusion with antimicrobial peptides or lysozyme that imparts remarkable antimicrobial properties (Barbosa et al. 2022; Ramos et al. 2010). Additionally, the fusion of CBMs with enzymes can alter catalytic performance and enzymatic characteristics, thereby finding applications in modifying multiple carbohydrate-active enzymes (Liu et al. 2022). Studies have shown that the absence of CBM resulted in a dramatic decrease in activities of amylase, SdG5A, from the marine bacterium *Saccharophagus degradans* under low temperature and high salt concentration (Ding et al. 2021). By using a flexible linker (GGGGS)2, Li et al. connected CBM9-2 to xylanase at its N-terminal region, yielding a fusion enzyme with an impressive 15.91-fold increase in specific activity (Li et al. 2022). Given the identification of HA-specific CBMs, it is plausible to explore their fusion with natural Hyals for enhancing catalytic performance and optimizing biochemical characteristics; thus expanding their application potential in bioengineering, drug delivery, tissue engineering and other fields (Oliveira et al. 2015; Shi et al. 2023).

## Conclusion and future outlook

**Fig. 5:**
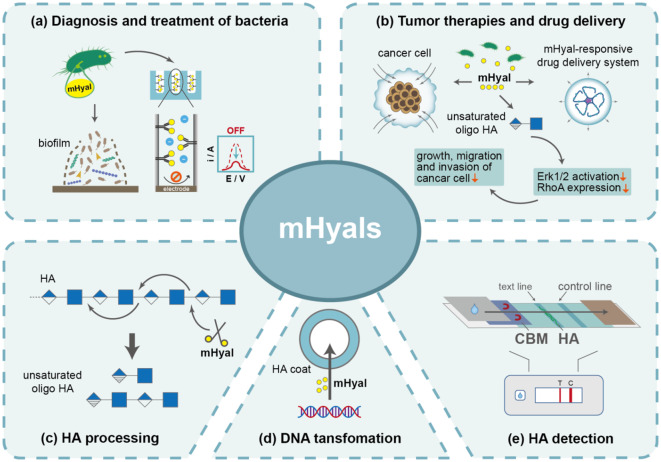
Function-inspired biotechnological applications of mHyals. **a** Applications of mHyals in diagnosis and treatment of bacteria. The mHyals are employed as antibacterial agents to disrupt biofilms. Additionally, mHyals can serve as reporter molecules for detection of bacterial infections. When interaction with the inner walls of the channels, mHyals impede the difussion of electroactive ions, leading to a reduction in voltammetric signal and thereby enabling differential pulse voltammetry-based detection of bacterial infections. **b** Applications of mHyals in tumor therapies and drug delivery. Engineered strains expressing mHyal can remodel the tumor stroma, synergistically enhancing the penetrability of anticancer agents and inducing tumor resolution. Unsaturated O-HAs metabolized by mHyals inhibits the growth, migration and invasion of cancer cells through disruption of Erk1/2 activation and RhoA expression. Moreover, mHyals specifically induce rupture of the drug delivery system, triggering the release of anti-cancer drugs. **c** mHyals act as scissors for HA depolymerization and functional oligo HA preparation. **d** mHyals facilitate DNA transfer in encapsulated cells. **e** CBM of mHyals is incorporated into lateral flow immunoassay for the identification of HA. The mobility of CBM exhibit a gradual decline in correlation with the increase in HA concentration within the samples. As the concentration of HA increases, there is a progressive reduction in the intensity of the T line, which could be completely abolished at excessively elevated levels of HA

The study of mHyals has been ongoing for over 70 years since its initial discovery as a “virulence factor”. Insights into the infection mechanisms of bacteria, fungi and bacteriophages have brought mHyals to forefront. Furthermore, the exploration of biological applications for mHyals is not as extensive compared to mammal and venom-derived Hyals. Based on current knowledge, we propose future research prospects in the following areas. Firstly, further exploration of mHyals resources is necessary. Despite numerous reports on mHyals, the efficiency and performance of current enzymes fall short in meeting the varied requirements for industrial applications. Therefore, it is imperative to acquire more promising mHyals as a prerequisite for advancing the industrial application of this enzyme. With the advancement of computational methodologies and structural biology, integrative approaches combining sequence-structure–function have demonstrated positive outcomes and hold great potential for significantly enhancing enzyme mining efficiency (Chen et al. 2023; Pardo et al. 2022; Tao et al. 2022). Secondly, construction of high-yield strains for efficient production of mHyals using genetic engineering strategies. Traditional strategies for optimization of regulatory elements such as promoters, ribosome binding sites and signal peptides have been used to enhance the production of mHyals (Zhang et al. 2023; Zhu et al. 2024). New strategies such as generating artificial promoters (Zhang et al. 2022a, b, c) and constructing quorum-sensing system (Su et al. 2023) might facilitate the production of target products. Thirdly, comprehensive research into the structure, catalytic mechanism, characterization of mHyals from different families. Currently, the structure and characterization of mHyals from PL8 and PL16 families have been elaborated; however, there is a dearth of comprehensive and systematic studies on mHyals from other families. In recent years, X-ray crystallography and cryoelectronic microscopy have facilitated the the acquisition of a plethora of three-dimensional enzyme structures, thereby aiding in the comprehension of the intricate relationship between the structure and function of enzymes (Maloney et al. 2022; Tao et al. 2022). By employing molecular dynamics simulations and quantum mechanical/molecular mechanics studies, a more comprehensive understanding of the molecular-level behavior of Hyals can be attained, which is pivotal for elucidating their biological functions and devising potential therapeutic strategies. Fourth, exploring the potential biological applications of mHyals. mHyals have been applied in medicine development, molecular biology, and industrial biotechnology (Fig. 5). The exploration of enzymes' potential applications will further enhance their utilization value and expand their prospects.

## Data Availability

Not applicable.

## Abbreviations

HA: Hyaluronan/hyaluronic acid
Hyals: Hyaluronan-degrading enzymes
mHyals: Microbial Hyals
GAG: Glycosaminoglycan
GlcA: Gluconic acid
GlcNAc: Acetylglucosamine
ECM: Extracellular matrix
LMW-HAs: Low-molecular-weight HAs
O-HAs: HA oligosaccharides
PL: Polysaccharide lyase
GH: Glycosyl hydrolase
∆HA2: Unsaturated disaccharides
spnHL: MHyals of *Streptococcus pneumoniae*
sagHL: MHyals from *Streptococcus. agalactiae*
TSβH: Triple-stranded β-helix
CBM: Carbohydrate-binding modules
GBS: Group B *streptococcus*
CSA: Chondroitin sulfate A
CSB: Chondroitin sulfate B
CSC: Chondroitin sulfate C
CSD: Chondroitin sulfate D
CSE: Chondroitin sulfate E
DS: Dermatan sulfate
PEG: Polyethylene glycol
Vc: Vitamin C
PUL_HA_: Polysaccharide utilization loci for HA
DHU: 4-Deoxy-L-threo-5-hexosulose-uronate
G-3-P: Glyceraldehyde-3-phosphate
MAC: Membrane attack complex
ITO/PET: Indium tin oxide//poly (ethylene terephthalate)
bHs-ST: *Salmonella typhimurium* Strain expressing functional mHyal
HB/Ag/g: HA/AgNPs/gentamicin nanocarrier
CPH: Polydopamine-modified chitin hydrogel
Chi: Chitosan
SL: Sodium hyaluronate-lauric acid
BMP2: Bone morphogenetic protein 2
TNT: Titanium nanotube
GET: Gene Electro-Transfe
HMW-HAs: High-molecular-weight HA
LPS: Lipopolysaccharide
LFIA: Lateral flow immunoassay
